# Phrenology

**Published:** 1823-03-01

**Authors:** 


*Observations on Phrenology, as affording a systematic View of Human Nature.* Edinburgh, Waugh & Innes—Ogle, Duncan, & Co. London; 1822.

Phrenology, from φρην mind, and λογος, is the term used by Dr. Spurzheim to denote a peculiar system of doctrines concerning the mind, founded on certain views of the physiology of the brain. The propriety of the term will become apparent, when we recollect, that the brain is the organ upon which the manifestations of the different mental powers depend; and that for every mental act, there must be a corresponding cerebral affection; and, therefore, that a correct exposition of the functions of this organ will necessarily imply a true theory of mind.

These doctrines have been taught for more than 20 years, at Vienna and Paris; but, it was not till 1816, when Dr. Spurzheim visited England, that general attention was attracted to them in this country. He delivered lectures upon the philosophy of his system, and gave demonstrations of the anatomy of the brain, by which, public curiosity was excited in an extraordinary degree, and much angry disputation occasioned. Established opinions received a shock, and all the partialities which accompany them were called into play to decry the merits of the new doctrines, on the one hand; while their novelty, and a certain degree of enthusiasm which inspired their advocates, shed a dazzling lustre around them on the other. Cool deliberation, always essential in forming a correct judgment of a new discovery, was thus excluded; and, after many pamphlets had been published and answered on the one side and the other, the subject appeared to fall into neglect, with its merits as undecided as when first introduced to the notice of the public.

After a silence of two or three years, however, the advocates of phrenology appeared again upon the field, and shewed, if possible, a stronger conviction of the truth of their opinions, and a more complete acquaintance with the doctrines themselves, as well as with the merits of the objections which had been stated against them. Mr. Combe's Essays on Phrenology led the way;—they appeared in November 1819, and their author testified strongly in favour of the truth of the new doctrines, and strenuously insisted on the necessity of enquiry into a subject which, in his opinion, so nearly affects the best interests of the human race. To this work, soon after succeeded the "Illustrations of Phrenology, by Sir G. S. M'Kenzie,"

in which the author gave portraits of remarkable characters, and compares their mental manifestations with the development of their brains. He contends, also, for the accuracy of the principles of phrenology. Several able criticisms next appeared in the pages of the New Edinburgh Review, in which the editor pledges himself that the doctrines are founded in nature. In 1820, Dr. Elliotson, in notes to the third edition of his Translation of Blumenbach's Physiology, gave a decided testimony to the truth and importance of the science.

In February 1820, a society was established in Edinburgh for cultivating and diffusing a knowledge of phrenology. A collection of skulls, casts, and busts, intended to exemplify the several organs and their combinations, was formed, and is every day receiving additions. This collection is open weekly to the inspection of the public. Meetings are held during the session, for discussing all subjects connected with phrenology, such as its application to elucidate the philosophy of mind, to education, legislation, medicine, and medical jurisprudence. The list of members includes gentlemen of talent and respectability of the three learned professions, besides artists and literary characters, and the numbers are on the increase. In 1821, Mr. Abernethy published "Reflections on Phrenology, addressed to the Court of Assistants of the College of Surgeons, London;" in which he treated it as the true system of the philosophy of mind, and recommended it to the special consideration and candid examination of medical men. About the same time, appeared Dr. Spurzheim's book on the Application of Phrenology to the Improvement of Education. In spring 1822, a society similar to that of Edinburgh, was instituted at Philadelphia, of which Dr. Physick is president, a gentleman who is well known to our readers. Among its members are to be found some of the most eminent men in law, medicine, and divinity, in that city. They have procured copies of all the casts in this country, and of the books in use among the phrenologists. Mr. Combe's Essays were reprinted under the direction of the society, and enriched with the anatomy which he had omitted. And, lastly, have appeared the "Observations on Phrenology," by the anonymous author, now before us, who considers it "as affording a systematic view of human nature."

When we find a rooted conviction of the truth of the new views, and a deep sense of their importance arising at this distance of time, in the minds of cool and able men, this circumstance itself affords a presumption, that there is something substantial at the bottom, whatever may have been added by fancy. The glare of fashion and novelty, which at first placed the structure in too dazzling a light to be clearly contemplated, has at last passed away, and it is perhaps only now that the subject comes properly under the candid and dispassionate consideration of the man of science, because now it comes before him stript of all foreign support, and resting entirely on the basis of its merits. The partialities and prejudices with which even the most vigorous minds are at times assailed, have had time, in a great measure, to disappear. The present moment is, therefore, particularly favourable for an examination of this long agitated question. Impelled by these considerations, and aware of the extreme importance to medicine and to philosophy, of the discovery of the functions of the brain, we have ventured to notice the publication at the head of this article, more with the view of seriously recommending a liberal and candid discussion of the merits of both sides of the question, as the most philosophical and sure way of ultimately arriving at the truth, than of attempting to deliver any decisive opinion of our own upon the subject.

We are led to desire this calm consideration the more, because hitherto, we conceive the weight of fact and argument and philosophy, to preponderate rather on the side of the phrenologists, than on that of their adversaries; not because we mean to assert that the opposite views are incapable of support, but that the opposition has been conducted in a way altogether unphilosophical; ridicule and vague assertion having been too unsparingly employed on the one side, while vigorous reasoning, and an appeal to Nature, have been constantly held out upon the other. The consequence has been, that although thousands boldly assert the whole of the doctrines to be imaginary, no person is to be met with, who is able to shew a clear and philosophical reason, why these ought to be disregarded; and it is a curious fact, well worthy of observation, that, except the late Dr. Gordon of Edinburgh, who fell, as it were, into a serious discussion inadvertently, and unintentionally, by his intemperate article in the Edinburgh Review, no author of note, either medical or metaphysical, has chosen fairly to grapple with the subject, and expose its errors; and of those, who have written, we do not know one, who has made good his objections in the judgment of impartial men, and replied satisfactorily to the answers given by the disciples of the new school. Dr. Roget, indeed, is another author of consideration, who has delivered an adverse opinion on their merits; but he appears rather as a historian of the doctrines, who sums up his account by delivering an opinion against them, than as a regular enemy who comes into the field to oppose and destroy them. On this account, we do not hold this gentleman as pledged even on the side which he has espoused, so as to stake his reputation on their futility, but view him as a critic open to conviction if he has pronounced an erroneous judgment. Many persons, no doubt, regard the doctrines as too ridiculous to merit a serious refutation, but we cannot subscribe to this opinion. The writings of Drs. Gall and Spurzheim themselves are worthy of a calm and philosophical refutation, if they contain erroneous views; but when other men of judgment, and not destitute of talent, come forward as supporters of their opinions; and not only so, but when societies are formed for their cultivation, we suspect that the tide of ridicule will soon begin to flow in an opposite direction, if those who patronize the established system, persevere in this supercilious treatment of their opponents. The contempt of the Chinese for the science and literature of Europe, does not arise from a more enlarged and comprehensive understanding in that nation; but it marks the extent to which ignorance and prejudice possess the mastery over their minds.

Other, and still stronger reasons, however, exist, why *serious enquiry* should be no longer delayed. The reader will bear in mind, that the general rejection of any new discovery affords no test whatever of its falsity. In proof of this, we have only to recollect the histories of the most important discoveries in medical or philosophical science. The celebrated Harvey, for his glorious discovery of the circulation of the blood, was long ridiculed and persecuted; his facts were neglected, and his theory met with almost universal condemnation. Hume mentions as a curious historical fact, that no physician, at that time, above 40 years of age, ever acknowledged his theory. Galileo was rewarded for his discovery of the motion of the earth, by persecution and imprisonment. Nor are these, by any means, solitary instances. Such occurrences are, in fact, but too common, and since such things have happened, we ought to be the more cautious, lest unwillingly, we add another to the list, even in this enlightened age.

In the next place, notwithstanding our almost total ignorance of the use of the brain, it has been universally regarded as one of the most important organs in the whole body, consequently, there has been no lack of talent or of zeal in endeavouring to ascertain its precise functions. The unceasing efforts of the most ingenious and profound philosophers, however, having, after a lapse of many hundred years, added so exceedingly little to our knowledge, we cannot but suspect that, if its precise functions are ever to be discovered, the modes of investigation hitherto in use, are not those, by which we shall be able to attain the end in view.

Physiologists are agreed, that the functions of any organ are not to be discovered by dissection alone; nevertheless, investigations of this kind ought to be conducted in such a way, as to afford, at least, a chance of discovering the *structure* of the organ, and the relation of its parts. In dissecting the brain, however, this rule has not hitherto been generally attended to. The organ is frequently examined, by slicing it horizontally, a mode of dissection, the use of which may be aptly illustrated, by supposing the same method applied in tracing the structure of the arm or thigh. What notion could be formed of the anatomy of these parts, by cutting circular slices through skin, muscles, blood-vessels, nerves, and bone, and minutely noting the appearances presented by each different section? How, then, can anatomists expect to succeed in discovering the structure of the brain, by slicing across the convolutions, instead of following the course of the fibres from the medulla oblongata to the farthest point of divergence to which they can be traced. The method of dissection, therefore, recommended by Drs. Gall and Spurzheim, appears to us well worthy of serious attention, in consequence of its own obvious merits alone, and still more so, seeing that it has met with the approbation of the French anatomists, whose opportunities of procuring brains in a proper state for examination, enable them to decide this point upon a more extensive experience than ours.

Although, however, the structure were perfectly ascertained, the functions would not be thereby disclosed; and as the founders of phrenology pretend to have discovered the functions also, two points naturally present themselves for our consideration, previously to forming a judgment upon their assertions. First; the means which they employed in their investigations: and, secondly, the coincidence or contradiction between the results obtained, and such facts in nature as are already known and established. If the means be philosophic and adequate, we may then listen to the conclusions. Should the results stand in opposition to established *opinions*, we ought not on this account alone to reject them, because established opinions may be erroneous; but as Nature is never inconsistent, if we find *ascertained facts* contradicting the phrenological views, we shall then have a good reason for treating them with disrespect.

First, then, with regard to the means. The operations of mind never come under our cognizance, but as connected with, and influenced by, certain changes in the state of the brain, for this organ is admitted, in all systems of philosophy, to be the medium, the *sine qua non;* by means of which, the mind acts upon, and is put in communication with, the external world. The immaterial principle eludes the research of our grosser senses, and we are obviously incapable of attaining a knowledge of the nature of mind as it exists in a disembodied state. All therefore that properly comes within the province of the physiologist or philosopher, is to ascertain the mutual influence of mind and organization upon each other, and the conditions of the organization required for the healthy operations of the different mental powers. Hitherto the extent of *certain knowledge* goes no farther than this, that a sound state of the brain is requisite for the sound manifestations of the mind ; but whether the whole brain is requisite for every single mental act, or, if not, what particular part is required for the due operation of any particular faculty has not been demonstrated. Dr. Gall advances still farther, and tries to prove that the latter view, viz. that of the plurality of faculties and organs is the true one, and this forms the fundamental principle of phrenology.

Now metaphysical philosophers, in investigating the phenomena of mind, have depended chiefly on the aid of consciousness for revealing the laws by which they are regulated, without attending to the effects of the organization. In doing so they appear to have taken too limited a view of the subject. The gradual and successive expansion of the infant faculties, keeping pace with the progressive perfection of the brain; the existence of idiotcy, and of insanity, and the every day phenomena of disease causing changes in the state of the mind, prove the constant and unceasing influence of the organic medium on the mental manifestations. The neglect of the influence of the organization appears therefore to have been one great cause of the constant failures which have hitherto occurred to metaphysicians in forming theories of the mind. Every mental act is performed by means of, and is influenced by, the organic medium, and yet, when in health, consciousness does not reveal to us even its existence. Its insufficiency, therefore, for unfolding the whole truth regarding the mind is obvious, and in consequence it is clearly an inadequate basis, on which to erect a complete system of mental philosophy. This conclusion appears the more sound, when we recollect that consciousness has never conducted two philosophers to precisely the same result; and that it varies in the same individual according to the state of his health, age, and other circumstances. By its aid we can discover only the successive conditions of our own mind upon the presentation of certain impressions, but because certain ideas or emotions arise or succeed each other in a certain order in one mind, and at a certain time, can we affirm, that such is the order in the minds of all men, at all times ? Certainly not; for the first person we meet with has a mind differently constituted from our own, and he denies the accuracy of our observations. So that by this means alone we can never discover the nature and number of the mental faculties, or the existence of the organ by which they act.

Physiologists being fully convinced that the functions cannot be discovered by dissection, have next supposed that by careful observation of the effects of injuries of this organ, we shall be able to ascertain the functions of its different parts; but we suspect that this method also is as inefficient as the others. Sir Everard Home, with the laudable view of throwing light upon a difficult subject, made a collection of upwards of fifty cases of cerebral injury, and noted carefully the effects produced, but these effects were all general, as coma, delirium, sickness, vomiting, and the like, without any constant or particular modification of one or several mental faculties ensuing from each particular lesion suffered by the brain. Such are the modes of enquiry hitherto in use.

In opposition to them, the phrenologists recommend the method of comparing the development of the head with the manifestations of particular faculties of the mind, in a state of health, and affirm that in cases where an individual propensity, sentiment, or intellectual power, is extremely vigorous, a certain part of the brain will be found very largely developed, while in other cases, where the same faculty is remarkably feeble, the same part will be found exceedingly small. The only rational objection which can be stated to this method, is that the skull does not indicate the size of the brain. The phrenologists reply to this objection that, admitting a certain degree of inequality or want of parallelism between the inner and outer tables to exist, it is so small (not exceeding the eighth part of an inch in the general case) as not to affect the result; because a large organ of cautiousness, or ideality, or love of approbation, for instance, will cause the skull to protrude a full inch more than a small organ will do, so that the inequality of one-eighth of an inch, even if it exists, is lost in the extent of the general difference. This also is a tangible statement; and if it be true, that persons of a very cautious character have an inch more of brain in a particular quarter than those who are very incautious, the fact is worth the knowing. Being solemnly asserted as a truth by persons of intelligence and reputation, it is the summit of absurdity to laugh at their assertion, but at the same time not dare to contradict it from observation. On the whole, therefore, we conclude that the common methods of investigating the connexion betwixt the mind and the brain, are not adequate to attain the ends in view, and that there is nothing in the phrenological mode of observation which ought to prevent us from putting it in practice.

In the next place, we may compare the alledged results of the phrenological method of enquiry with several well ascertained natural phenomena, and mark the coincidence or opposition between them.

The phrenologists affirm, that a plurality of faculties and organs exist, and that one of them may be diseased, deficient, or more than usually vigorous, without affecting the state of the others, just as the organ of vision, of taste, or of touch, may be defective in one individual, while that of hearing, or of smell, remains in its usual state. The doctrine which stands opposed to this is, that the whole brain constitutes but one organ, and that every part of it is employed in every mental act. Now let us apply the two theories to several well ascertained physiological and pathological phenomena, and mark which of them quadrates most exactly with positive experience. The following facts are too well ascertained to admit of dispute. We find some individuals, who, from birth, are deficient in some one or more of the internal faculties of the mind, as in the case of partial idiots.

"It is remarked," says Fodéré, in his Traité du Goitre et du Cretinisme, p. 133, "that by an inexplicable singularity, some of these individuals (cretins) endowed with so weak minds are born with a particular talent for copying paintings, for rhyming, or for music. I have known several who taught themselves to play passably on the organ and clavecin; others who understood, without ever having had a master, the repairing of watches, and the construction of some pieces of mechanism." He adds that these powers could not be attributed to the intellect, "for these individuals not only could not read the books which treated of the principles of mechanism, *mais ils etaient deroutès lorsqu'on en parlait,* et ne se perfectionnaient jamais."

Pinel speaks of a female idiot who had the most astonishing propensity to imitate whatever she heard or saw done. She repeated whole sentences, but without attaching any idea to them. He distinctly admits the fact that individuals exist who from birth possess a limited capacity for receiving certain kinds of ideas, and an utter incapacity for all other kinds; or in other words, who possess one or more faculties of the mind to a limited extent, and appear to be deprived of all the others. Every writer on insanity, as Rush, Haslam, Esquirol, admits the partial development of certain powers of the mind in idiots, and Rush in particular not only alludes to the powers of intellect, but also to the partial possession of the moral faculties. Some idiots, he observes, are as remarkable for correct moral feelings, as some great geniuses are for the reverse. One never utters an intelligible sentence, because he does not comprehend it, although he can repeat the words automatically, like a magpie or parrot, while another not only understands the words of his mother tongue, but even delights in speaking. One is all good nature and complaisance, another quarrelsome and mischievous. One is pious, and another obscene. Dr. Rush mentions one who was remarkable for his religious feelings, and we have frequently seen an idiot who could never connect two sentences, except when engaged in devotion, to which he had a natural tendency. He was able to repeat a prayer verbatim, after hearing it two or three times. Fodéré calls these "inexplicable singularities." No one will suspect him of favouring phrenology by reporting cases which have no existence, and as they exist, we really agree with him in saying that they are truly inexplicable on the general supposition of the mind manifesting all its powers by the medium of a single organ. The phrenologist, on the other hand, says that to him they offer no such difficulty, for supposing it proved that each faculty manifests itself by the medium of a separate organ, it is as easy to conceive that one of these organs may be defective from birth, with or without a corresponding deficiency in all the others, and of course with a deficiency in the faculty connected with that organ, as it is to conceive that the organ of hearing may be unfit for performing its function, while those of sight, taste, smell, and touch, may either remain unimpaired, or may be impaired to a less degree. In the case of the external senses, he maintains, we never hesitate in assigning the cause and seat of the deficiency to the organ which executes the function of the diseased sense. We never refer it to a difference in the constitution of the immaterial principle. Then why should we not also refer defects in the internal senses or faculties to an imperfection in the state of the organ of the particular faculty in fault? It would certainly be far more philosophical and consonant to Nature to do so, than to say that God had created souls of idiots and souls of men of genius.

The phrenologists assert that, upon examination, these differences are found sufficiently accounted for by the defective state of the organization; and that partial idiots, for example, although they manifest one or more faculties more powerfully than others, yet seldom manifest any with the energy of a sound mind. Now, Pinel and many other writers agree in saying that idiots from birth are generally possessed of very deficient brains, taking them as a whole, and this observation we have had an opportunity of confirming from our own experience, both in this country, and among the cretins in Switzerland, who are particularly alluded to by Pinel. Some idiots, no doubt, have large heads, but they are either distended by water or otherwise diseased. Farther, these individuals differ in the *qualities* as well as *quantities* of their mental endowments. Were the brain *as a whole* the organ of mind, and its general small size the cause of idiotcy, it follows that they ought to be idiotic to the same extent *in all* their faculties, or they ought to be capable of manifesting every faculty exactly to the same extent, which is not the case, for the same individual sometimes possesses some faculty to a considerable degree while he is extremely defective in others. To say then that one organ manifested all these faculties is much the same as if we said that the eye is the organ of hearing as well as of vision, or that all the five senses executed their office by means of a single organ, because the difference between the faculties possessed and those deficient in the former case is as great as in the latter, only in the first the division of the organs is more concealed from our external senses. Another set of cases, with which we are well acquainted, is that in which one or more faculties of the mind are diseased, while the others remain sound, or the state known by the name of monomania. Pinel, Esquirol, Haslam, Cox, Rush, and, in short, every author on insanity, speaks of the partial affection of the mental faculties as a thing of every day occurrence, and the very adoption of a particular name to designate that state is in itself sufficient evidence of the existence of such partial insanities. Esquirol believes that in monomania some affection of the sentiments alone exists, without disease of the intellectual faculties, while in mania the intellect is deranged. Here again the difficulty recurs of accounting for these cases on the supposition of one organ executing all the functions of the mind, while on the hypothesis of the existence of a plurality of organs, one serving each faculty, no embarrassment appears. The phrenologists, indeed, affirm that, without being intended, monomaniacal affections have received different names according to the nature of the faculty most excited by disease. Thus erotomania and nymphomania, according to them, are the consequences of diseased action of the cerebellum, or organ of the sexual propensity, and many surgical cases on record, particularly several related by Baron Larrey in his History of the Campaigns, and by Serres in his late work, are strongly corroborative of the accuracy of the function assigned to this organ. Melancholy is another term in general use, and in its common acceptation is said to denote morbid activity of the organ of cautiousness, sometimes modified by that of conscientiousness, as when the patient believes himself to have committed crimes, and done actions which were calculated to excite remorse; sometimes, by that of veneration and hope, as in the case of religious melancholy. "Folie raisonnante," it is affirmed, denotes either a natural state of the organs of intellectual faculties, while those of the propensities or sentiments are diseased, or, an increased energy of the same organs of intellect from morbid excitement. The leading characteristics of these cases are, that the patient reasons justly and often with great acuteness and logical accuracy, but his data are false; thus, during disease, a manifesting talent, sometimes general, sometimes partial, in a degree which is quite unnatural to him in a state of health, and which ceases with the disease. Rush speaks of the reasoning powers, and the talent for mechanics manifesting themselves in a most extraordinary degree during a paroxysm of insanity. Dr. Perfect, in his Annals, mentions having seen similar cases. He particularizes an uneducated girl, employed in manual labour, who, during the paroxysm, expressed herself in beautiful verse. Pinel speaks of a man who, during the paroxysm, possessed uncommon power of mind, and discoursed with infinite ease and judgment on the important events of the day, and those of the revolution at that time at its height.

Injuries of the head, as well as fevers, and other acute diseases also, are well known sometimes entirely to change a person's natural disposition, although the intellect remains as powerful as before. Rush says that some have shewn a degree of honour and integrity during and after disease, to which they were formerly strangers; while others have lost their usual habit of veracity, their verbal memory, or their sweetness of temper, by the occurrence of acute diseases, although their intellect was not impaired. Such facts the phrenologists affirm to be inexplicable, except on the supposition of a plurality of organs. In the next place, when we witness the gradual and successive development of the different powers of the infant mind, taking place exactly in the order in which the wants and situation of the individual seem to require them, and when we know that the faculties, which are essential for observation, arrive at maturity long before the age of manhood, while the reasoning powers may be said to be only then beginning to appear in their proper state, we are again embarrassed by the notion of a single organ; for a general organ cannot be both *deficient* and *sufficient* at the same moment. Farther, on looking around us we perceive at a glance the greatest differences in the dispositions and mental capacities of different individuals even from the most tender to the most advanced age, among the learned and intelligent as well as among the illiterate and stupid—one is naturally of an open and generous nature, another shy, timid, and suspicious—one is fearful of attracting that notice in which another delights, and for which he pants—one shews a great talent for music, another for drawing, modelling, or calculating—one easily repeats long passages which he may have heard or read, another can never master a line, however willing—one is quick but superficial, skimming over the surface of things; another is slow, but profound and fond of abstract reasoning—one manifests great genius for some particular pursuit, in which he easily excels, although his genius is thwarted or discouraged by the mistaken kindness or prejudice of ignorant and bigoted parents or preceptors; and at last, when free from restraint, it becomes the ruling passion of his soul—another, aided by all the power of riches, and the best masters which the most affectionate parents can procure, nevertheless fails in arriving even at mediocrity. All these cases are admitted to exist, but by philosophers they have generally been attributed to the effect of external circumstances, as education, &c. but we believe, without sufficient reason; for we observe every gradation of intellectual capacity, from the lowest pitch of idiotcy up to the genius of a Bacon, and that often in circumstances exactly similar. In the case of manifest idiotcy no one disputes that the cause is organic. Tracing, then, one degree of mental endowment after another, from the lowest up to the highest, at what point does or can the cause of it cease to be organic? and if it be organic, how can one organ be so powerful in executing some of the mental acts, and so feeble in manifesting others at the same stage of life, and in the same external circumstances? These facts and the production of talent or exaltation of intellectual energy by disease are sufficient to shew that genius and dispositions must be born with us, before they can be called into action by external circumstances, and consequently the cause on which they depend must belong to the body.

It must be admitted, that these and similar facts are extremely difficult of explanation, except on the phrenological principle of plurality of faculties and organs, and we had almost said, that some of them, such as the cases of erotomania, religious melancholy, "folie raisonnante," afford at least strong probability of the correctness of the division of the faculties, as presented by Drs. Gall and Spurzheim. At all events the occurrence of partial idiotcy and partial insanity, the gradual and successive development of the different faculties as the child advances from infancy to manhood, the development of certain kinds of talent by disease, and which disappear with the disease, and the change of disposition produced by injuries and other morbid affections of the brain, appear to us amply sufficient to prove that the fundamental principle of each particular mental faculty depending for its due operation on the proper constitution and development of the particular organ of that faculty, is in strict conformity with many of the phenomena of mind as observed in the states of health and of disease, and is therefore deserving of the most serious examination and refutation previous to its rejection.

Considerations similar to the foregoing have long since induced many philosophers strenuously to maintain the impossibility of all the faculties operating by a single organ, and the existence of several independent powers of the mind manifested by an equal number of independent organs. Their conclusions were supported by the same kind of facts and observations, which led many physiologists from an early period to contend for the independent existence of two kinds of nervous fibres in certain nerves, although inclosed in the same sheath, and so closely interwoven as to set at defiance the most patient efforts of the anatomist, who attempted to distinguish them by dissection. Sensation was sometimes found to remain in a palsied limb, when the power of voluntary motion was lost, and *vice versa.* The same fibre could not, they said, be both sound and diseased at the same moment; and therefore, although anatomy could not demonstrate the distinction, yet facts having occurred which precluded the possibility of a single nerve being in opposite states at the same time, they were in a manner forced to admit the existence of two kinds of fibres. The correctness of the conclusion has lately been successfully demonstrated by the experiments of Mr. Charles Bell, in London, and of Magendie in Paris, to which we alluded in our last number. The latter gentleman appears to have conducted his investigations in ignorance of the discovery of Mr. Bell, so that both having arrived at nearly the same results, more confidence can be placed in the accuracy of their experiments. This fact of two kinds of nerves running undistinguishably in a common sheath, although executing separate functions, ought to make us pause before denying the *possibility* of the division of the brain into several organs, on the ground that anatomy does not reveal the existence of any boundary or line of demarcation between them. We mention this here, because some have laid considerable stress on it as an objection to phrenology.

It appears, then, to be more philosophical and more in unison with the phenomenon of mind, as observed both in health and in disease, to hold that the mind is possessed of a number of primitive and distinct powers or faculties, depending for their operation on the healthy condition of a corresponding number of organs, of which the brain appears to be a congeries; than to hold that the mind is merely a general power capable of being directed as external circumstances require, and manifesting all its powers by the medium of a single organ.

The accuracy of the facts and observations made by Drs. Gall and Spurzheim must, however, be refuted or confirmed by experience alone, and we have pursued this line of argument merely to shew that their mode of enquiry is not only reasonable, but highly philosophical and worthy of attention —that if the accuracy of their observations is confirmed by those of future enquirers, it will advance the philosophy of mind with a certainty and success far greater than have hitherto attended the efforts of its most devoted cultivators. Certainly there appears little deserving of ridicule or contempt in such views of this highly important subject. We wish therefore to excite the spirit of enquiry, that the foundation and successive additions to the new science may be carefully examined, and that their correspondence or disagreement with nature may be ascertained and made known before we definitively either adopt or reject it.

It was the philosophic character, and uniform consistency of the new doctrines, and their apparent conformity to nature, which led the author of the work before us, as well as Mr. Abernethy, to regard them as probable, highly important, and well worthy of the notice of medical enquirers. The author before us appears to be a person of very considerable talent, and he expressly mentions that he is not yet a convert to the truth of phrenology, not having made observations so as to qualify himself for deciding upon the accuracy of the facts acted upon by the phrenologists. He states that he came to the examination of the subject with a mind prejudiced against it, as a thing in itself absurd, and of no consequence even if true. When, therefore, he declares himself to have been much surprized and interested in discovering a new and apparently sound system of the philosophy of mind, where he expected to find only a collection of visionary and absurd speculations; when the powerful mind of an Abernethy believes the new system to be that of the true philosophy of mind, merely from an examination of the ground work, and a consequent perception of its consistency and harmony with Nature, we cannot possibly believe that such doctrines can be so very absurd and heterodox as they have sometimes been represented. Mr. Abernethy, in an especial manner, recommends them to the attention of the medical profession, as probable in themselves, and teeming with the most important results. Our author, in his preface, says, that he was one of the "many who treated the doctrines of phrenology with disregard, if not with contempt and derision. His opposition did not proceed from conviction arising from any examination, but rather from an ignorance of what they truly were, and a general impression of their being absurd, heterodox, and irreconcilable to the principles of true philosophy."

Some articles in favour of phrenology, which appeared in the New Edinburgh Review, excited his attention, and induced him to examine the standard works on phrenology. In the course of this enquiry, it appeared to him that the prevailing opposition to the new doctrines was at least as much owing to misapprehension of their importance as to any doubt of their truth. He therefore conceived that—

"If it could be shown that this system (of philosophy) is a reasonable one, and that it is conformable to analogy, and to the order of nature in other instances—that it accounts for the phenomena in a perspicuous and beautiful manner—that every part of it tends to some useful end, and that the whole is well connected and consistent with itself; such a demonstration, could it be accomplished, would afford a kind of internal evidence of the truth of the system, which would go far to remove the *aversion*, with which it seems at present to be regarded by many of the most intelligent and scientific in this country," and "the following pages contain the substance of an attempt to elucidate the subject in this way."

In another place (p. 46) he remarks—

"I am sensible I have not treated the subject in the manner a phrenologist would have done. Taking his stand upon the high ground of facts, and firm from the conviction arising from experience, he will probably feel no addition to his faith from any arguments drawn from other sourses, and may regard as needless any attempts to support the system by probable reasoning, or what he may consider fanciful and wire-drawn anologies. But there may be minds so constituted as to be affected by such arguments and analogies, and which require to be invited to the study of the facts by such means."

In this observation we are inclined to agree with our author, and anxious to excite enquiry and a candid examination, we can recommend his "Observations" to the attention of those who, aware of the value of truth from whatever quarter it comes, only require a rational motive for proceeding to the investigation. With the same view, we have taken some pains to shew that the mode of enquiry now held out to us is both rational and promising, and that we may follow it with less hesitation, seeing that those hitherto in use have availed us so little. Had the author joined the testimony of observation and experience to that derived from reasoning, so as to give a decided opinion from self-conviction of the truth of the new doctrines, no doubt he would have produced a deeper impression. But, even as they are, his "Observations" along with Mr. Abernethy's "Reflections" are valuable as testimonies of the true nature and character of a system so much misrepresented. From a decided convert the public would expect admiration of doctrines which he considered as founded in nature, but from one who still doubted, no such thing could be expected, and yet he, even in doubt, could not refrain from adding his tribute to the beauty, harmony, and consistency, which characterize the whole.

Our author first discusses the probability of the brain being an assemblage of different organs, each having for its function the operations of an individual faculty. He says—

"If different parts of the brain are destined to different purposes, where is the absurdity of supposing, that certain separate portions of the brain are more intimately connected with, and more closely subservient to, different individual functions of the mind, than any other part? If this be so, it may appear to us a very curious and wonderful provision; but it is no more absurd or inconsistent with reason, than that different organs are appropriated to the use of the different senses—that the eye is connected with, and subservient to, the sense of vision, the ear to that of hearing, and the tongue to that of taste. The only difference is, that in the one case the organs are more open to observation, their configuration is more mechanical, and more obvious to our gross and imperfect powers of observing—but in the principle itself, that the different powers may have different portions of the brain assigned to them, connected with and subservient to them, and by means of which they act and manifest themselves, there is no absurdity whatever. *It may perhaps not be true*—that is a different matter, and must be decided by observation and experience; but it is quite conformable to reason and analogy to say, *that it may be so.*" 8.

He then proceeds to discuss the nature of the powers of the mind to which the different organs are respectively subservient, and the situation of the organs as given by Drs. Gall and Spurzheim. In looking over the list of faculties in order to try if he could reconcile it to reason and analogy, he could observe no order or connexion between them.

"The whole presented to me a rude appearance, quite different, as I then thought, from what is commonly found in nature. After a more attentive consideration, however, light began to dawn upon me, and beginning to consider the faculties in a certain way, and to group them after a certain order, the whole gradually formed themselves before me into a system of surprizing symmetry, and, like the disjointed parts of an anamorphosis when seen from the proper point of view, collecting themselves under one elegant design, delighted me with the appearance of that very order and beauty which I would before-hand have expected to find in them."

He subsequently examines the situation and functions of each organ individually, and adds some good remarks on the effects of each on the general character, and in different states of society, and he thinks the probability of the localities being correctly marked, is increased from the consideration of the intimate connexion existing between those which mutually require the assistance of each other. He is also disposed to admit the existence of the number of faculties discovered, seeing that all appear necessary for the explanation of the phenomena. In the "peculiar positions that are assigned to them (the organs) in this system, there are circumstances of connexion and mutual relation to which we could hardly have attended, and which seem to surpass any effort of mere human ingenuity."

The harmonious junction of the organs, the beautiful adaptation of the faculties to each other and to the phenomena of mind as observable in every state in which it exists, are, he says, far too remarkable, and the coincidences are far too numerous and exact, to have occurred by chance.

"As soon would a number of letters shaken out of Swift's laputan machine fall of themselves into the order of a scientific treatise, as that the names of thirty-three faculties, put down at random, should compose a complete and well combined scheme of the human mind, such as this appears actually to be. The inference is, I think, irresistible, either that the scheme which appears so well arranged has been invented by the ingenuity of Drs. Gall and Spurzheim; or, if they actually proceeded in the manner in which they tell us, and formed it piecemeal by a gradual and patient examination of facts, that the harmony and systematic junction of these scattered members forms a very strong presumption (to say no more) in favour of the accuracy of their separate observations, and of the system being truly founded in nature."

The system is so different from any formerly stated to the world, that he thinks it impossible it should ever have occurred to the mind of any individual in any other way than that in which it did occur to Drs. Gall and Spurzheim. And that it did occur to them in the manner alluded to is proved by the early publications and plates including comparatively few of the thirty-three faculties now given. The discovery of the connexion of a few qualities of the mind with particular portions of the brain could never lead the founders of the science to suspect that in reality this was the beginning of the discovery of the true philosophy of mind, a philosophy which could not be overturned like preceding theories, because founded on facts in nature instead of being the offspring of an ingenious hypothesis. Accordingly it seems only to have been after ascertaining the existence of the greater number of the present list of faculties, that the veil appeared to withdraw itself, and a system of philosophy to occupy the place of what appeared before unconnected, though curious and interesting facts.

Our author observes in addition that—

"Had Drs. Gall and Spurzheim sat down with the purpose of constructing a system from their own imagination, it is next to morally impossible that they could have contrived one which harmonized so completely with itself, and with the actual state of the human faculties, and the uses to which these faculties are subservient. This is a problem which has puzzled the most eminent philosophers of ancient and modern times; and all attempts to solve it have hitherto been fruitless, so as almost to entitle us to conclude that its accomplishment is beyond the reach of human ingenuity. If, then, these gentlemen had actually succeeded in inventing a system like this, which affords a key to the mental constitution of man, and a facility of accounting for the diversities of human intellect, and human character, far surpassing any other system that ever appeared—supposing it to be, as all former systems have been, entirely hypothetical—it would entitle the authors of it to rank as philosophers along with the highest names which have ever adorned the annals of the world."

This our readers will think would be strong language even from a convert; but coming from one who has only a half acquaintance with phrenology, not having observed the facts on which it is alledged to rest, and who from his own statement came to the enquiry with his mind prepossessed against it, what are we to think? surely there must be a greater air of probability about the system when taken as a whole, than one would be apt to suppose from the disjointed statements and anecdotes with which the public have been long entertained. The author before us is clearly of this opinion, since he has published for the sake of stimulating to farther enquiry.

"I am not aware," says he, "that any such connected view of this system has been given as the above, or that the mutual relation, and dependence of its different parts have been brought forward exactly in the light in which I have endeavoured to place them. The argument may not strike every one as strongly, but to me I own it appears to have considerable weight. It goes, at least, the length of inclining me to examine the facts in which the system is said to be founded, and of removing any positive dislike to it, or hardness of believing it on reasonable evidence."

Writing merely to excite enquiry into the truth of a system which he esteems highly important and interesting to mankind, by proving its reasonableness and conformity with whatever is ascertained to be fact in the science of mind, our author has confined himself to arguments drawn from the state of the faculties during health alone. We have extended the argument to the state of the mind during disease, believing that if the truth is discovered, it will be found consistent with the phenomena of mind in every condition, whether of health or of disease. And for the same reason of wishing to excite enquiry, and of not vouching for the truth of the facts from his own experience, the author does not enter upon any of the objections which have been made against them. Had he been either a decided believer, or a decided opponent, he never could have passed them over in silence. This article, intended for the introduction of the subject to the notice of our readers, has already extended to too great a length to admit of our examining the objections in detail. Generally speaking, none of them appear to be opposed to the principles of the science, but all to hinge, more or less, on the difficulties of the enquiry.

